# Bullying Behaviors and Stress (Acute and Perceived) among Undergraduate Nursing and Midwifery Students: The Moderating Role of Gender and Academic Majors

**DOI:** 10.3390/healthcare12161588

**Published:** 2024-08-09

**Authors:** Abdulaziz Mofdy Almarwani, Atallah Alenezi, Mohammed Almutairi, Fahad M. Alhowaymel, Naif S. Alzahrani, Hanan F. Alharbi, Abdulaziz Fahad Abaoud

**Affiliations:** 1 Department of Psychiatric Nursing, College of Nursing, Taibah University, Medina 42353, Saudi Arabia; 2 Department of Nursing Sciences, College of Applied Medical Sciences, Shaqra University, Shaqra 11961, Saudi Arabiafalhowaymel@su.edu.sa (F.M.A.); aabaoud@su.edu.sa (A.F.A.); 3 Department of Medical Surgical Nursing, College of Nursing, King Saud University, Riyadh 12372, Saudi Arabia; 4 Department of Medical Surgical Nursing, College of Nursing, Taibah University, Medina 42353, Saudi Arabia; 5 Maternity and Pediatric Nursing Department, College of Nursing, Princess Nourah bint Abdulrahman University, P.O. Box 84428, Riyadh 11671, Saudi Arabia

**Keywords:** nursing, midwifery, students, bullying, stress, undergraduate, Saudi Arabia

## Abstract

Background: Nursing and midwifery professionals’ well-being may be affected by various factors, including the bullying of new nurses. Purpose: To analyze the relationship between bullying behaviors, perceived stress, and acute stress among undergraduate nursing and midwifery students in Saudi Arabia, as well as the moderating role of gender and academic majors in this relationship. Method: A cross-sectional correlation design was used, and data were collected from 322 undergraduate nursing and midwifery students enrolled in four major nursing universities in Saudi Arabia. Results: Educational level, environment, and personal attacks had a significant negative impact on perceived and acute stress (social and non-social), while hostility toward academic achievement and direct negative behaviors had significant positive impacts on perceived and acute stress (social and non-social). Female nursing students reported a stronger impact of bullying behavior on their perceived stress, while male students reported a greater impact on social and non-social stress. Nursing students reported a stronger impact of bullying on perceived stress, while midwifery students reported a greater impact on social and non-social stress. Discussion: Nursing educators should raise awareness about the harmful effects of bullying and emphasize the importance of creating a safe, supportive learning environment.

## 1. Introduction

Nursing education is plagued by constant attrition among newly graduated nurses and increasing nurse shortages. Several factors affect the well-being of nursing and midwifery professionals, including bullying behavior directed toward them [1]. Bullying has been an issue in nursing and midwifery for many years and is an increasingly common cause of nurse retention and recruitment difficulties [2]. Bullying among nursing and midwifery students is a widespread and alarming problem in both the theoretical and practical domains of the nursing profession. The prevalence of bullying can present significant obstacles to the well-being and professional growth of aspiring healthcare professionals who must complete demanding coursework and training. To provide a supportive learning environment and encourage the mental and emotional resilience required to join the next generation of healthcare workers, it is imperative to acknowledge and address the complex realities of bullying in this population.

The incidence of bullying has various ramifications for the nursing and midwifery professions. Bullying experienced by students in these professions might increase their chances of developing mental health problems, including stress, which can affect their educational progress [3] and their perception of nursing and other healthcare professions. Additionally, bullying encountered in the course of students’ enrollment in undergraduate programs may negatively impact their psychological well-being [4].

Bullying extensively causes role stress [5], occupational stress [6], post-traumatic stress [7], and acute, chronic, and episodic acute stress [8,9,10,11]. Furthermore, the literature reveals three major sources of stress: (i) academic stressors, including heavy academic loads, taking examinations, and long studying hours [12,13,14]; (ii) clinical stressors, including taking care of patients, fear of making mistakes, bullying, interactions with clinical staff and faculty, and deficiency of professional nursing knowledge and skills [12,13,14]; and (iii) personal stressors, including financial problems, difficulty in balancing personal and academic life, job responsibilities, and maintaining part-time employment positions while studying [13,14,15].

According to Aljohani et al., nursing students experience more stress than students in other healthcare specialties [13]. A systematic review revealed that more than 60% of nursing students suffer from stress [16]. The stress levels of nursing students can be significantly affected by bullying, which makes an already challenging academic and clinical setting even less welcoming [17]. Stress among nursing students can contribute to burnout and anxiety and can even increase students’ chances of becoming depressed [18]. In some cases, stress becomes overwhelmingly problematic and severe, causing students to develop acute stress disorders [19]. 

The investigation of bullying behavior dynamics among nursing and midwifery students has revealed the possible influence of gender as a moderating factor [20,21]. In educational and clinical contexts, bullying can have a significant impact that varies with its prevalence, the specific types of behavior, and how it is perceived. Moreover, according to research, bullying behavior patterns may differ among male and female nursing students, impacting how events are reported, understood, and handled [22,23]. To our knowledge, however, few studies have examined the relationship between bullying and the prevalence of stress and acute stress among undergraduate nursing students. Moreover, this relationship has yet to be studied among undergraduate nursing and midwifery students in Saudi Arabia. Previous research has also emphasized the need to study the role of gender in the relationship between bullying behavior and stress [23,24,25,26]. Furthermore, having both nursing and midwifery students as samples of this study, as well as their occupational and learning differences [27,28,29,30], it would be interesting to examine how different the impact of bullying on stress is between nursing and midwifery students. 

Since education precedes practices, educators in these fields must understand the relationship between bullying and the prevalence of stress and acute stress among students so that future interventions to minimize or eliminate such acts can be applied. Similarly, students need to understand the effect of bullying on stress to allow them to derive coping strategies to deal with it. Studying this phenomenon will give educational institutions an opportunity to improve the educational experience for students before they enter a workplace in which bullying may be anticipated. 

In the present study, the researchers investigate the relationships between bullying behavior, perceived stress, and acute stress among undergraduate nursing and midwifery students in Saudi Arabia. The researchers also examined the role of demographic variables in the underlying relationship between bullying behavior, perceived stress, and acute stress. Hence, the researchers set out to answer the following research questions:

1. Are there relationships between bullying behavior, perceived stress, and acute stress among undergraduate nursing and midwifery students?
2. Does gender moderate the relationships between bullying behavior, perceived stress, and acute stress among undergraduate nursing and midwifery students?
3. Does academic major (nursing vs. midwifery) moderate the relationships between bullying behavior, perceived stress, and acute stress among undergraduate nursing and midwifery students?

### 1.1. The Literature Review 

#### 1.1.1. Bullying Behavior among Students

Several studies have been conducted on the topic of bullying behavior among nursing students [16,31,32]; however, there are limited studies on midwifery students. Fernández-Gutiérrez and Mosteiro-Díaz conducted an integrated systematic review of the available literature on this general topic and found 31 studies that highlighted the prevalence, perception, associated profiles, importance, causes, and effects of bullying behaviors among nursing students, as well as strategies for coping with harassment adopted by nursing students in difficult circumstances [23]. In a recent study by Amini et al., a significant number of nurses were found to be victims of workplace bullying, with more than half of the nurses reporting moderate emotional exhaustion, depersonalization, and impeded academic achievement; in addition, bullying was found to have a strong association with burnout [33]. In addition, Celdrán-Navarro et al. recently conducted a systematic review of counter-bullying nursing interventions and discovered several autonomous and interdisciplinary interventions to address and prevent bullying [34]. 

Bullying behavior comes in many forms, such as being isolated from the educational environment such as being let alone during breaks, and direct negative behaviors, such as being exposed to physical violence or verbal abuse. Also, being attacked due to their academic achievements, such as being forced to perform a job that will negatively affect a student’s confidence, or attacks on their personality, such as being questioned on student’s honesty and reliability [4]. 

Regarding the isolation of students from the educational environment, Pigozi and Bartoli [35] highlighted that isolation of the students from a group is a form of indirect bullying, which is hard to recognize due to its less evident nature. Birks et al. [36] also explained that as a sign of isolation, students intentionally leave the ward, making the bullied student feel like they are not part of the health care team and never speak to them during tea breaks and recess.

Regarding attacks on academic achievement, Seibel and Fehr [37] reported that students felt they received bad grades and were burdened with assignments and additional responsibilities for punishment rather than educational purposes and did not receive acknowledgment for their clinical or academic accomplishments. Acimis and Tekindal [38] found that prevention of self-expression and communication is another form of bullying, which causes a reduced level of personal achievement. 

In terms of attacks on personality, Mohd Halim et al. [39] revealed that a lower level of humility and sincerity—a characteristic of personality—caused a higher level of depression through bullying. Birks et al. [36] and Courtney-Pratt et al. [40] highlighted that eye-rolling and talking about students behind their backs are forms of non-verbal bullying behavior that reflect the passive–aggressive personality experienced by nurses.

Lastly, in the attribution of direct negative behavior, Birks et al. pointed out that bullying behavior involves injustice incidents, unfair treatment, and public humiliation [41]. In another study, Birks et al. [36] ascribed verbal, racial, physical, and sexual abuse as types of bullying behavior, which, in particular, are recognized as negative behavior. Amoo et al. [42] also stated that sexual harassment and verbal abuse are forms of bullying behavior, which are characterized as negative behavior demonstrated by senior medical professionals.

#### 1.1.2. Perceived and Acute Stress among Students

Perceived stress can be defined as a student’s perception of stress, whereas acute stress [43], or short-term stress, can be defined as stress that persists for a period (from a week to a month) after a traumatic incident [44]. Most existing research has focused on post-traumatic stress disorder and perceived stress among nursing students, nursing staff, and doctors [13,16,18,45,46,47]. However, few studies have specifically addressed acute stress, particularly among nursing and midwifery students in Saudi Arabia. Acute stress tends to cause intrusions, arousal, altered mood, and reactivity and motivates individuals to persistently avoid stimuli associated with the traumatic event [19]. Furthermore, acute stress disorder significantly causes psychological distress [19] and negatively affects career planning and career retention [48].

#### 1.1.3. Role of Gender and Academic Major in Relationship between Bullying and Stress

The cultural differences between Saudi Arabia and Western or even other Middle Eastern countries, especially in being more restrictive for women [49,50], may make the relationship between bullying and stress more distinct for men and women, which has yet to be studied. Furthermore, nursing and midwifery students differ in occupational roles [27], qualifications and status in hierarchical structures [28], professional quality of life [29], clinical learning environment, educational approach of the clinical instructors, and resulting differences in students’ perceptions toward clinical learning environment [30]. Considering these differences, there has been a lack of information regarding how bullying, as an antecedent of stress, may differ between undergraduate nursing and midwifery students.

#### 1.1.4. Theoretical Framework

This study is guided by the transactional theory of stress. According to this theory, workplace bullying has a strong association with perceived stress [47]. The theory highlights that workplace bullying is committed by individuals exposed to highly stressful situations and explains that workplace bullying threatens people’s basic need to perceive the world as controllable and predictable [51]. Hence, workplace bullying results in perceived stress among nurses, which, in turn, leads to high turnover and long-term sick leave. Additionally, Berry et al. (2016) emphasized that perceived stress is higher among registered nurses who are exposed to workplace bullying behaviors daily [31]. Relatively speaking, nursing is a highly stressful and demanding occupation in a stressful environment that can deteriorate interpersonal relationships, leading to hostile or bullying behaviors [52,53]. 

The learning environment plays a vital role in the educational journeys of nursing and midwifery students. Bullying limits and degrades students’ experiences, deprives them of obtaining necessary skills and knowledge and shows a negative image of the nursing profession [37]. Due to the instructors’ mistreatment, undervaluation, and delays in students’ academic learning progress, students can feel shame, anger, doubt, and self-blame, which worsen their psychological health, intensify their stress levels, and adversely affect their academic performance [3]. Hence, nurse educators must not tolerate any form of bullying within the learning setting for any reason. When subjected to bullying, students experience heightened levels of stress, which ultimately hinders their ability to absorb and retain knowledge effectively. 

## 2. Materials and Methods

### 2.1. Study Design

The researchers adopted a cross-sectional correlational research design to collect data from Saudi nursing and midwifery students. The data were collected through a questionnaire within a single time period and analyzed to determine the relationship between bullying behavior, perceived stress, and acute stress.

### 2.2. Sampling

Through purposive sampling, the researchers recruited 322 undergraduate nursing and midwifery students enrolled in bachelor’s programs in Saudi Arabia. The primary reason for choosing purposive sampling was the ease of reaching nursing students and the large population of nursing students who fit the inclusion criteria—namely, being enrolled in four-year nursing bachelor’s programs at four major universities in Saudi Arabia (both men and women) and willing to participate in this study. To ensure explicit willingness to participate in the survey, information about the purpose and nature of this study was included, and an assurance of confidentially and anonymity was given in the questionnaire. 

### 2.3. Sample Size Estimation 

G-Power was used to estimate the sample size [54]. The parameters used to estimate the sample size were as follows: effect size (f2) = 0.04; alpha = 0.05; power = 0.80; and number of predictors = 4. This method yielded a total sample size of 302 participants.

### 2.4. Setting

An online survey was distributed to undergraduate nursing students enrolled in nursing and midwifery bachelor’s programs in the eastern and central regions of Saudi Arabia.

### 2.5. Research Instruments

The questionnaire consisted of four parts: (i) demographic characteristics; (ii) bullying behaviors; (iii) acute stress; and (iv) perceived stress. The demographic characteristics included age, gender, marital status, university name, academic year, and academic major. The questions on bullying behaviors, acute stress, and perceived stress were adopted from previously established measures/questionnaires, as discussed below.

#### 2.5.1. Bullying Behaviors

Bullying behaviors were measured using the Bullying Behaviors in Nursing Education scale developed by Cerit et al. [4]. This scale is used to measure bullying behaviors experienced during nursing education in academic environments. It asks about four factors: (i) isolation of students from the educational environment (4 items); (ii) attacks on academic achievements (4 items); (iii) attacks on personality (6 items); and (iv) direct negative behaviors (4 items) [4]. The tool uses a six-point Likert scale (0 = never experienced; 1 = experienced a few times a year; 2 = experienced a few times a month; 3 = experienced a few times a week; 4 = experienced once a day; and 5 = experienced a few times a day) [4]. One of the items on the scale is “Not being wanted in the study group related to the school or internship”. These factors had Cronbach’s alpha values of 0.70, 0.78, 0.78, and 0.72, respectively, indicating that the scale was reliable. 

#### 2.5.2. Acute Stress Questionnaire

Acute stress was measured using Kent et al.’s College Student Acute Stress Scale [44]. This scale is used to measure acute stress specific to the college student experience. It consists of two factors: (i) social stress (i.e., stress caused by social life, recreation, and relationships with friends, family, professors, and classmates) and (ii) non-social stress (i.e., stress caused by factors other than socialization, such as financial problems, schoolwork, etc.) [44]. Social and non-social stresses have six and seven items, respectively, which are measured on a five-point Likert scale (0 = no stress; 1 = a little stress; 2 = some stress; 3 = a lot of stress; and 4 = constant stress) [44]. One of the questions in the scale is “In the last month, how often have you felt nervous and ‘stressed’?” These factors had Cronbach’s alphas of 0.83 and 0.79, respectively, indicating good reliability.

#### 2.5.3. Perceived Stress Scale

Perceived stress was measured using the Perceived Stress Scale developed by Cohen et al. [43]. This scale is used to measure the degree to which situations in a person’s life are appraised as stressful. The scale has 10 questions, measured on a five-point Likert scale ranging from 0, indicating “never”, to 4, indicating “very often” [43]. One of the questions in the scale is “In the last month, how often have you felt nervous and ‘stressed’?” The scale had a Cronbach’s alpha of 0.76, indicating good reliability.

### 2.6. Statistical Analysis

Descriptive statistics, including the mean and standard deviation, were used to describe the age of the respondents, while frequency distribution was used to describe the frequency and percentage of their demographic characteristics. Means and standard deviations were also used to describe the variables under study, i.e., bullying behaviors, acute stress, and perceived stress. Covariance-based structural equation modeling (CB-SEM) was conducted to analyze the relationships between the factors of bullying behaviors, acute stress, and perceived stress among undergraduate nursing students. Here, CB-SEM analysis was used to test an existing theory (i.e., transactional theory of stress) in a new context. Furthermore, it was flexible to use in this study, as there were more than five observations per indicator, data were normally distributed, and there were at least three items per construct [55]. To achieve better model fit, measurement models for the three main variables (i.e., bullying behavior, perceived stress, and acute stress) and seven sub-dimensions of the main variables were assessed separately through confirmatory factor analysis, and the model with higher model fit (i.e., measurement model of seven sub-dimensions) was selected for analysis. Moderation analysis was conducted to analyze the moderating role of gender and academic majors in the relationships between bullying behavior, acute stress, and perceived stress. 

### 2.7. Human Ethics and Consent 

Approval for this study was obtained from the institutional review board at the College of Applied Science at Shaqra University (ERC_SU_20220097). All students submitted signed, written, informed consent electronically. Students who took part in this study were provided with clear information about the goals of the research and were informed that their participation was voluntary and that all participants had the option of withdrawing from this study at any time. To ensure the privacy and confidentiality of the participants’ information, the data were accessible only by the primary investigator and co-investigators.

## 3. Results

### 3.1. Demographic Characteristics

A total of 322 nurses and midwifery students participated in this study. Most respondents were female (68.9%), studied nursing as their major (77%), were currently enrolled in their second year of the nursing degree (42.2%), and attended classes around four to six hours per week (36.6%). The average age of the nurses was 20.2 years, with a variability of 1.31 years (Table 1). 

### 3.2. Confirmatory Factor Analysis

Confirmatory factor analysis was conducted to analyze the standardized factor loadings, reliability, and validity of the variables under study (Table 2). The results indicated that the standardized factor loadings of one item from “isolation of students from the education environment”, one item from “attack on academic achievement”, four items from “perceived stress”, one item from “social stress”, and two items from “non-social stress” were 0.323, 0.456, −0.259, 0.026, 0.070, 0.118, 0.301, 0.316, and 0.249, respectively, which were all lower than the standardized factor loading of 0.60 suggested by Awang [56] and Afthanorhan et al. [57]. The Cronbach’s alpha (α) values of all variables ranged from 0.746 to 0.875, while the composite reliability values of all variables ranged from 0.761 to 0.870, which is higher than the threshold level of 0.70 suggested by Hair et al. [58] (Table 2). This indicates that our values were reliable. The average variance extracted (AVE) of all variables ranged from 0.507 to 0.516, which is higher than the threshold of 0.50, indicating an acceptable level of convergent validity (Table 2). The square-root values of the AVE of all variables were higher than the inter-construct correlation values, indicating acceptable discriminant validity (Table 2). Lastly, the measurement model had an excellent fit for all measures (CMIN/DF = 2.009 < 3.00; CFI = 0.913 > 0.90; GFI = 0.855 > 0.85; TLI = 0.902 > 0.90; IFI = 0.914 > 0.90; RMSEA = 0.056 < 0.08; SRMR = 0.0528 < 0.08). To assess the correlation between variables under study, Pearson’s correlation was conducted and found that all dimensions of bullying behaviors, acute stress (i.e., social and non-social), and perceived stress were significantly associated with each other (*p* < 0.001) (Table 3). 

### 3.3. Structural Equation Modeling

**Figure 1:**
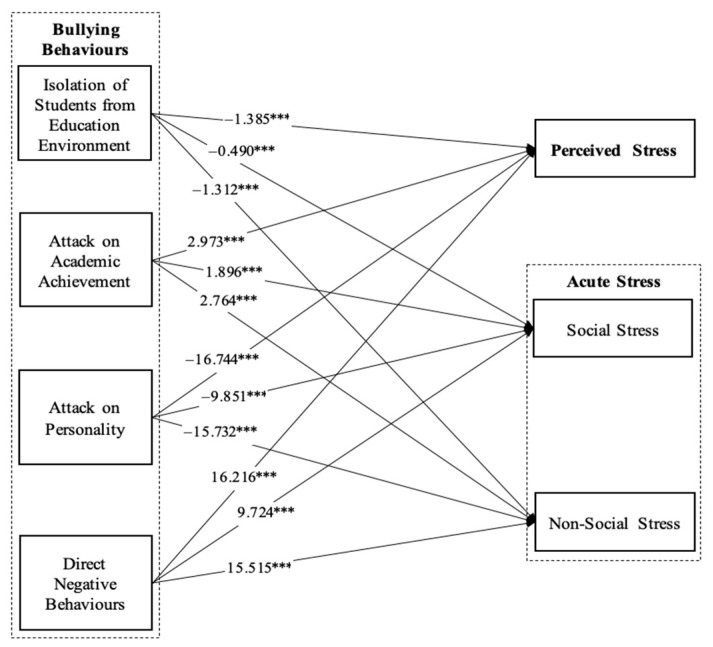
Structural Model. Note: *** *p* < 0.001.

Structural equation modeling was conducted to assess the relationship between specific aspects of bullying behaviors (i.e., isolation of students from the educational environment, attacks on academic achievement, personal attacks, and direct negative behaviors), acute stress (i.e., social and non-social stress), and perceived stress (Figure 1). The structural model had an excellent fit for all measures (CFI = 0.921 > 0.90; NFI = 0.921 > 0.90; IFI = 0.922 > 0.90; RMSEA = 0.069 < 0.08; SRMR = 0.072 < 0.08) and accounted for 93.5%, 81.9%, and 48.6% of the variance in perceived stress, social stress, and non-social stress, respectively. The results indicate that the isolation of students from the educational environment had a significant negative impact on perceived stress (β = −1.385; *p* < 0.001), social stress (β = −0.490; *p* < 0.001), and non-social stress (β = −1.312; *p* < 0.001). Moreover, personal attacks had a significant negative impact on perceived stress (β = −16.744; *p* < 0.001), social stress (β = −9.851; *p* < 0.001), and non-social stress (β = −15.732; *p* < 0.001). However, attacks on academic achievement had a significant positive impact on perceived stress (β = 2.973; *p* < 0.001), social stress (β = 1.896; *p* < 0.001), and non-social stress (β = 2.764; *p* < 0.001). Moreover, direct negative behaviors had a significant positive impact on perceived stress (β = 16.216; *p* < 0.001), social stress (β = 9.724; *p* < 0.001), and non-social stress (β = 15.515; *p* < 0.001).

**Figure 2:**
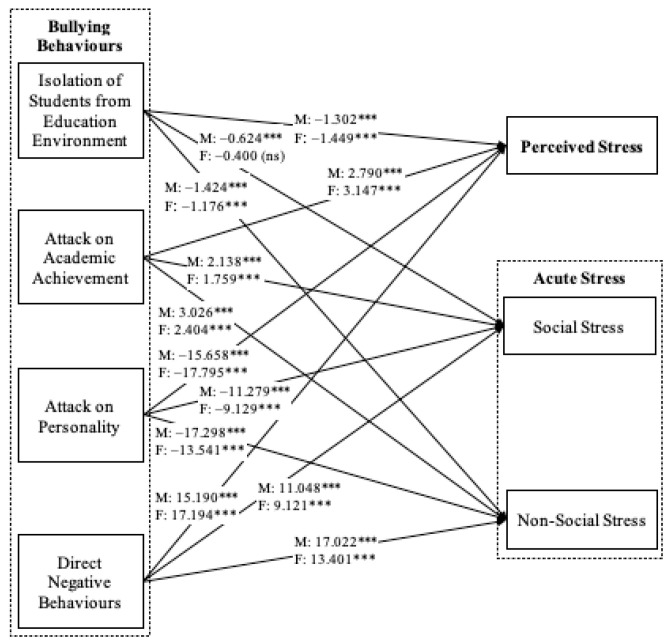
Structural Model—Moderating Effect of Gender. Note: M = Male, F = Female, *** *p* < 0.001, ns = not significant.

**Figure 3:**
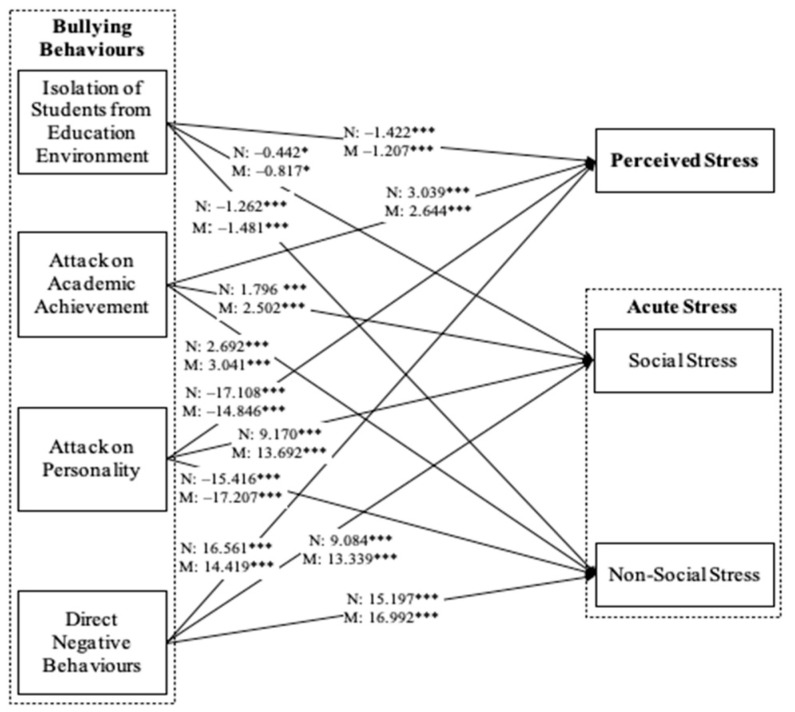
Structural Model—Moderating Effect of Study Major. Note: N = Nursing, M = Midwifery, * *p* < 0.05, *** *p* < 0.001, ns = not significant.

Beyond testing for direct relationships between bullying behaviors, perceived stress, and acute stress, structural equation modeling using moderation analysis was also conducted separately to examine the moderating role of gender and academic major in these direct relationships (Figure 2 and Figure 3). Each model determined how the relationships between bullying behaviors, perceived stress, and acute stress differed between men and women (Figure 2) and between nursing and midwifery students in terms of magnitude and direction (Figure 3). The results indicate that gender and academic major were significant moderators in the relationship between the specific aspects of bullying behaviors, perceived stress, and acute stress (gender: Δχ^2^ = 25.376, *p* < 0.05; academic major: Δχ^2^ = 30.688, *p* < 0.001). In terms of gender, all four types of bullying behaviors had a stronger impact on perceived stress among female nursing students, but they had a greater impact on social and non-social stress among male nursing students (Figure 2). In terms of academic major, all four bullying behavior factors had a stronger impact on perceived stress among nursing students but had a greater impact on social and non-social stress among midwifery students (Figure 3).

## 4. Discussion

This cross-sectional correlational study was conducted to analyze the relationships between bullying behaviors, perceived stress, and acute stress among undergraduate nursing and midwifery students in Saudi Arabia. The researchers also examined the moderating roles of gender and academic majors within these relationships. This study revealed significant relationships between bullying behavior, acute stress, and perceived stress. In particular, increased isolation of students from the educational environment and attacks on personality decrease acute (i.e., social and non-social) and perceived stress among nursing and midwifery students. In other words, students who keep themselves away from the school environment, friends, and social groups are less likely to experience stress. Our results reveal that even if students are humiliated and questioned on their honesty, reliability, and body language in public, they do not seem to have higher levels of stress. Having reduced stress helps students overcome feelings of disappointment, anger, and nervousness and, hence, efficiently deal with the difficulties they face. Such students can also better manage their financial, health, and transportation problems and are less concerned with their relationships with friends, family, classmates, and teachers. This negative relationship is consistent with the findings of Noack and Linden [59] and Mosanya [60]. Noack and Linden [59] highlighted that the resulting stress caused by public humiliation did not meet the criteria of post-traumatic stress disorder, although it caused a feeling of embitterment to a great extent. Mosanya [60] indicated that dispositional grit or passion, perseverance, and mental durability toward academic achievements prevented students from experiencing stress due to social isolation during the COVID-19 pandemic. However, the negative relationship in this study contradicts the findings of Yildirim et al. [61] and Bergin and Pakenham [62]. Notably, bullying victims are often found to have neurotic personalities, meaning that they are highly prone to anxiety and depression and are moderately vulnerable to stress as well [63]. Yildirim et al. (2007) highlighted that isolation from the workplace and attacks on personality are the most common bullying/mobbing behaviors suffered by nursing teaching staff, and responses to such behaviors are largely determined by tiredness, perceived stress, and headaches [61]. Bergin and Pakenham (2015) concluded that social isolation has a moderate to strong relationship with stress, depression, and anxiety among law students [62]. 

This study also found that an increase in attacks on academic achievement and direct negative behaviors increased perceived and acute stress (i.e., social and non-social) among nursing and midwifery students. In particular, students who have limited self-expression, are never trusted for their competencies, are forced to perform jobs, and are overburdened with work feel higher levels of stress. Similarly, students who had practical jobs or were exposed to verbal or physical abuse faced higher levels of stress, causing increased disappointment, anger, and nervousness, made them un-socialized and rendered them unable to manage their financial, health-related, and transportation problems. This result is consistent with the findings of Berry et al. [31], Finchilescu et al. [64], Mohamed [65], Kousar et al. [32], and Abdelaziz and Abu-Snieneh [3]. Finchilescu et al. [64] highlighted that the effects of bullying behavior on nursing students included stress-related conditions, such as intimidation, anxiety, tension, and self-doubt, as well as impeded clinical learning [64]. The consequences of bullying behavior include hypertension, depression, and post-traumatic stress [65], which directly affect the professional skills of nursing students and their patients’ safety [32]. Abdelaziz and Abu-Snieneh [3] found that student exposure to bullying and maltreatment was related to poor mental health and perceived stress among nursing students [3]. Berry et al. [31] found a significant increase in perceived stress, anxiety, and post-traumatic stress in people who suffered frequent bullying at work [31]. 

This study discovered a stronger impact of bullying behaviors on perceived stress and a greater impact on social and non-social stress among male students, except for the impact of isolation of students from the education environment, which had a stronger impact on perceived, social, and non-social stress among female students. This indicates that men who face extensive bullying in the forms of attacks on personality, attacks on academic achievement, and direct negative behaviors perceive higher levels of stress and suffer from short-term stress due to loss of relationships with family, friends, classmates, or teachers and because of financial, transportation, and health-related problems. Our findings contradict those of Chatziioannidis et al. [66] and Östberg et al. [67]. Chatziioannidis et al. [66] found that females were bullied significantly more than men and had higher levels of psychological distress, while Östberg et al. [67] concluded that being bullied was associated with greater perceived stress among both males and females.

The present study revealed a stronger impact of bullying behaviors on perceived stress among nursing students but a stronger impact on social and non-social stress among midwifery students. This indicates that bullying causes stress differently among nursing and midwifery students, as the caused stress in each group has distinct attributes, i.e., perceived stress for nursing students and social and non-social stress for midwifery students. These results are consistent with those of Birks et al. [41] and Capper et al. [21]. Birks et al. [41] found that half of the nursing students they studied had experienced bullying in the preceding 12 months and subsequently felt greater stress and anxiety. Capper et al. [21] explained that midwifery students are affected by the high-stress environment of the maternity unit and other stressors, such as high workloads, staff shortages, poor skill mixes, high levels of complex and medicalized care, and poor treatment by managers. They suggested that these organizational factors promote bullying behaviors directed toward midwifery students. 

### 4.1. The Role of Nurse Educators

Nursing curricula should consider and protect students’ psychological well-being. Building a curriculum that meets students’ needs—for instance, by managing and reducing bullying—is important. Nurse instructors have the authority to integrate initiatives against bullying into educational programs, offering instruction on interpersonal communication, conflict management, and assertiveness abilities. By promoting open dialogue, fostering empathy, and demonstrating professional conduct, nurse educators can enable nursing students to identify and confront instances of bullying.

Nurse educators are of the utmost importance in the effort to decrease bullying among nursing students. As guides and facilitators of education, they have the potential to foster a culture centered around reverence, understanding, and acceptance within the nursing education setting. Nurse educators can enhance awareness regarding the negative consequences of bullying and stress the significance of establishing an environment that is supportive and secure for learning purposes. Moreover, nurse educators have a responsibility to empower nursing students to overcome bullying. Empowerment can increase students’ capacity to deal with bullying in a professional and efficient manner.

When creating curricula and educational interventions designed to combat bullying, nurse educators should give special consideration to the idea of empowering nursing students. Nurse educators are responsible for fostering an inclusive and supportive environment that encourages growth and learning without the risk of harassment or intimidation. By doing so, we can empower nursing and midwifery students to thrive academically and develop into compassionate professionals equipped to provide exceptional care to their patients.

### 4.2. Theoretical Implications

This study was built on the idea presented in the transactional theory of stress that workplace bullying has a strong association with perceived stress. The findings of this study expanded this theory by explaining the forms of bullying behaviors that caused stress as well as by specifying the types of stress that were found to be affected by bullying behavior. Furthermore, this study explained how bullying could be a series of negative interpersonal acts that could configure high levels of stress, as reflected by the transactional theory of stress [68]. Additionally, this theory only established the relationship between workplace bullying and perceived stress. However, this study further develops the notion of this theory by ascertaining the relationship between bullying and acute stress. Furthermore, this study applied the underlying theory on nursing education and confirmed the same results, enhancing the theoretical implications of the transaction theory of stress.

### 4.3. Study Limitations and Suggestions for Future Research

This study has several limitations. First, the researchers used a correlational research design, as the researchers aimed to determine the relationships between bullying behaviors, perceived stress, and acute stress. However, the researchers did not analyze the prevalence of bullying behaviors, perceived stress, or acute stress among nursing or midwifery students. Future research can be conducted on this topic. Second, the researchers only focused on bullying behaviors as a determinant of stress and did not study the impact of related factors (e.g., workload, self-reflection, personality, and organizational culture) that could increase stress or mediate or moderate the underlying impact of bullying behavior on perceived and acute stress among undergraduate nursing students. Future research should expand on the existing model through aspects such as mediators and moderators. Third, the relationship between bullying and stress could be significant due to the self-reporting of students on stress and bullying behavior, as there are individuals who look for empathy and inhibit desperation to attract attention to themselves, which makes them vulnerable to bullying and increases their level of stress unrelated to bullying. Future research can explore the relationship between bullying behavior and stress in clinical environments, where bullying is stimulated in a controlled setting, and clinical professionals and psychologists can estimate and measure the stress of nursing midwifery students. Fourth, the researchers studied the impact of bullying behavior on stress for undergraduate nursing and midwifery students only. Future studies can explore this relationship between bullying and stress for other medical professionals, such as doctors and physicians, to increase the generalizability of this study. Additionally, similar studies can be conducted in other countries to confirm the significance of relationships between bullying and stress, as studied in the current study. Such replication studies can confirm the unexpected negative relationship between the isolation of students from the education environment and attacks on personality on perceived, social, and non-social stress, which is rarely found in the current literature on this topic. Finally, future studies can explore specific interventions to prevent bullying behavior and effective coping strategies to endure bullying and, consequently, stress. 

## 5. Conclusions

This study reported that, overall, bullying behaviors had a significant impact on perceived and acute stress among undergraduate nursing students. In particular, the isolation of students from the educational environment and personal attacks had a significant negative impact on social, non-social, and perceived stress, while attacks on academic achievement had a significant positive impact on social, non-social, and perceived stress. This study also indicated that bullying had a stronger impact on perceived stress among female students but a stronger impact on social and non-social stress among male students. Finally, this study found that bullying behaviors had a stronger impact on perceived stress among nursing students but a greater impact on social and non-social stress among midwifery students. Hence, gender and academic major moderate the relationships between bullying behavior, acute stress, and perceived stress among undergraduate nursing students.

## Figures and Tables

**Table 1:** Demographic Characteristics (N = 322).

Measure	N	M	SD
Age (years)	322	20.2	1.31
**Measure**	**N**	**%**	
Sex			
Male	100	31.1	
Female	222	68.9	
Study Major			
Nursing	248	77	
Midwifery	74	23	
Current Nursing Year			
First Year	18	5.6	
Second Year	136	42.2	
Third Year	109	33.9	
Fourth Year	52	16.1	
Internship Year	7	2.2	
University			
University 1	47	14.6	
University 2	149	46.3	
University 3	50	15.5	
University 4	76	23.6	
Time Spent Studying per Week			
Less than an hour	11	3.4	
1–3 h	72	22.4	
4–6 h	98	30.4	
7–9 h	62	19.3	
More than 10 h	79	24.5	

**Table 2:** Confirmatory Factor Analysis-Reliability & Validity.

Items	M (SD)	SFL	α	CR	AVE	MSV	MaxR(H)
*Bullying Behaviours*							
*Isolation of students from the education environment*			0.746	0.761	0.516	0.100	0.766
Not being wanted in the study group related to the school or internship	0.48 (0.93)	0.762					
Not being accepted to the group of friends	0.56 (1.02)	0.675					
Intentionally leaving the environment when you enter an environment	0.63 (1.21)	0.715					
*Attack on academic achievement*			0.800	0.803	0.507	0.353	0.812
Limited self-expression	1.19 (1.49)	0.700					
Not being trusted in the competence related to lectures	0.91 (1.29)	0.712					
Being forced to do a job that will negatively affect your self-confidence	0.75 (1.31)	0.786					
Constantly assigning tasks over the capacity	1.54 (1.42)	0.642					
*Attack on personality*			0.844	0.841	0.518	0.078	0.862
Talking in a humiliating and degrading style	0.59 (1.22)	0.625					
Questioning your honesty and reliability	0.64 (1.11)	0.684					
Being scolded loudly in public	0.50 (0.99)	0.831					
Using degrading mimics or body language	0.39 (0.96)	0.805					
Talking bad or unfounded behind you	0.54 (0.94)	0.627					
*Direct Negative Behaviours*			0.807	0.804	0.508	0.057	0.814
Making practical jokes	0.39 (0.90)	0.773					
Being exposed to verbal or behavioural sexual implications	0.32 (0.76)	0.639					
Mild violence to intimidate	0.36 (0.84)	0.762					
Being exposed to physical violence	0.29 (0.85)	0.668					
*Perceived Stress Scale*			0.875	0.870	0.530	0.465	0.885
Being upset because of something that happened unexpectedly	1.70 (1.25)	0.689					
Feeling unable to control important things in life	1.78 (1.26)	0.851					
Feeling nervous and ‘stressed’	2.47 (1.24)	0.784					
Unable to cope with all things that you had to do	1.60 (1.15)	0.699					
Feeling angered because of things that were outside of your control	2.15 (1.26)	0.626					
Feeling difficulties piling up so high that you could not overcome	1.95 (1.28)	0.696					
*Acute Stress Scale*							
*Social Stress*			0.860	0.860	0.507	0.412	0.866
Relationships with friends	0.67 (1.04)	0.716					
Relationships with parents	0.60 (1.08)	0.668					
Relationships with professors/teachers	0.86 (1.06)	0.728					
Recreation activities	0.53 (0.93)	0.650					
Social life	1.00 (1.11)	0.802					
Relationships with classmates	0.58 (0.93)	0.699					
*Non-Social Stress*			0.806	0.806	0.510	0.465	0.808
Schoolwork	1.50 (1.25)	0.736					
Financial problems	1.31 (1.25)	0.689					
Health problems	1.19 (1.36)	0.802					
Transportation problems	1.75 (1.30)	0.691					

**Table 3:** Mean, Standard Deviation, & Correlation between Study Variables.

		M	SD	1	2	3	4	5	6	7
1	Isolation of Students from Education Environment	0.560	0.860	1.00						
2	Attack on Academic Achievement	1.10	1.090	0.707 **	1.00					
3	Attack on Personality	0.530	0.822	0.666 **	0.725 **	1.00				
4	Direct Negative Behaviours	0.340	0.667	0.659 **	0.645 **	0.992 **	1.00			
5	Perceived Stress	1.94	0.971	0.377 **	0.665 **	0.315 **	0.273 **	1.00		
6	Social Stress	0.710	0.786	0.674 **	0.815 **	0.609 **	0.570 **	0.710 **	1.00	
7	Non-Social Stress	1.44	1.024	0.441 **	0.684 **	0.458 **	0.426 **	0.763 **	0.817 **	1.00

** *p* < 0.001.

## Data Availability

The corresponding author can provide the datasets utilized and/or analyzed in the present study upon a reasonable request.

## List of Abbreviations

CB-SEM	covariance-based structural equation modeling
α	Cronbach’s alpha
CR	composite reliability
AVE	average variances extracted
CFI	comparative fit index
NFI	normed fit index
IFI	incremental fit index
RMSEA	root mean square error of approximation
SRMR	standardized root mean square residual
